# Supplementary material for: What Country, University, or Research Institute, Performed the Best on Covid-19 During the First Wave of the Pandemic? Bibliometric analysis of scientific literature – analysing a ‘snapshot in time’ of the first wave of COVID-19
Source: Ann Data Sci. 2022 Jun 28;9(5):1049–67. doi: 10.1007/s40745-022-00406-8 (PMC9243965; doi:10.1007/s40745-022-00406-8)
Supplement: Supplementary file 1 — Supplementary Material 1 [file 40745_2022_406_MOESM1_ESM.docx]

# Response to reviewers

We are very grateful for the helpful comments from the reviewers. In this file, we include the responses to reviewers feedback, and we remain available for making any further corrections that might be requested by the reviewers.

Comments from reviewers and response with a description of new editing and corrections

Comment 1: A. Since this journal is about Data Science, I suggest you take this revision seriously by adding some discussion about data science in introduction section. You also should cite the following papers of AODS and another journal in some places of your paper carefully to make your revision, and list them in the references: 
1.	Khakharia A, Shah V, Jain S, Tiwari, A, Daphal P, Warang M, Mehendale N (2021) Outbreak Prediction of COVID-19 for Dense and Populated Countries Using Machine Learning. Ann Data Sci 8: 1–19.
2.	Li J, Guo K, Herrera Viedma E, Lee H, Liu J, Zhong Z, Gomes L, Filip FG, Fang SC, Özdemir MS, Liu XH, Lu G, Shi Y (2020) Culture vs Policy: More Global Collaboration to Effectively Combat COVID-19. The Innovation, <https://doi.org/10.1016/j.xinn.2020.100023>.
3.	Liu Y, Gu Z, Xia S, Shi B, Zhou X, Shi Y, Liu J (2020) What are the Underlying Transmission Patterns of COVID-19 Outbreak? An Age-Specific Social Contact Characterization.  EClincialMedicine, Vol. 22, 100354, May 01.
4.	Shi Y (2022) Advances in Big Data Analytics: Theory, Algorithm and Practice, Springer, Singapore.
5.	Olson DL, Shi Y (2007) Introduction to business data mining. McGraw-Hill/Irwin, New York.
6.	Shi Y, Tian YJ, Kou G, Peng Y, Li JP (2011) Optimization based data mining: theory and applications. Springer, Berlin.
7.	Tien JM (2017) Internet of things, real-time decision making, and artificial intelligence. Ann Data Sci 4(2): 149–178.

Corrections and editing in response to comment 1:

New section 1.2 has been added in the data mining and data science section and the old section is included as a sub-section 1.2.1. The text includes all references listed above. Copied text:

## ‘Discussion on data science

Data science consists of (1) design for data; (2) collection of data; and (3) analysis on data; and can be described as ‘synthetic concept to unify statistics, data analysis and their related methods’ [1]. Data science practitioners apply integrated techniques to analyse real-world big data problems. Some of the integration concepts of big data analytics and/or data science include ‘multi-criteria optimization for learning, expert and rule-based data analysis, support vector machines for classification, feature selection, data stream analysis, learning analysis, sentiment analysis, link analysis, and evaluation analysis’ [2]. Data mining practical applications in various fields (e.g., financial analysis, credit management, health insurance, network intrusion detection, internet services analysis) are often enhanced with optimisation techniques, such as (1) Support Vector Machines for Classification; (2) LOO Bounds for Support Vector Machines; (3) Unsupervised and Semi-supervised Support Vector Machines; (4) Feature Selection via *l_p_* - Norm Support Vector Machines; (5) Multiple Criteria Programming; etc. [3]. Data science is strongly represented in business data mining [4], for real-time decision making with a combination of internet-of-things (IoT) and artificial intelligence (AI) technologies [5]. More recently, the Covid-19 pandemic has been analysed with various data science tools, e.g., 1 for outbreak prediction of the top 10 highly and densely populated countries, using Auto-Regressive Moving Average [6]; e.g., 2 for ‘age-specific social contact characterization.. of the underlying transmission patterns’ [7]. The data mining in this article is more closely related to ‘culture vs policy’ [8] with the aim of promoting more global collaborations to combatting global pandemics with technological solutions.’

Comment 2: B. Precisely follow the author guidelines of the journal: <https://www.springer.com/journal/40745/submission-guidelines#Instructions%20for%20Authors_References>
All of references must be listed in order of the usage by numbers. You need to cite each reference with [number] in appropriate places of the paper. The style of the citations of the journal papers, book, book chapter, conference proceedings and so on can be referred as the below:

Citation requirement:
Reference citations in the text should be identified by numbers in square brackets. Some examples:
1. Negotiation research spans many disciplines [3].
2. This result was later contradicted by Becker and Seligman [5].
3. This effect has been widely studied [1-3, 7].

Corrections and editing in response to comment 2.1: This referencing format is followed. We used Mendeley referencing software.

Reference list
The list of references should only include works that are cited in the text and that have been published or accepted for publication. Personal communications and unpublished works should only be mentioned in the text. Do not use footnotes or endnotes as a substitute for a reference list.
The entries in the list should be numbered consecutively.
Journal article
Gamelin FX, Baquet G, Berthoin S, Thevenet D, Nourry C, Nottin S, Bosquet L (2009) Effect of high intensity intermittent training on heart rate variability in prepubescent children. Eur J Appl Physiol 105:731-738. <https://doi.org/10.1007/s00421-008-0955-8>
Ideally, the names of all authors should be provided, but the usage of “et al” in long author lists will also be accepted:
Smith J, Jones M Jr, Houghton L et al (1999) Future of health insurance. N Engl J Med 965:325–329 
Article by DOI 
Slifka MK, Whitton JL (2000) Clinical implications of dysregulated cytokine production. J Mol Med. <https://doi.org/10.1007/s001090000086>
Book
South J, Blass B (2001) The future of modern genomics. Blackwell, London
Book chapter
Brown B, Aaron M (2001) The politics of nature. In: Smith J (ed) The rise of modern genomics, 3rd edn. Wiley, New York, pp 230-257
Online document
Cartwright J (2007) Big stars have weather too. IOP Publishing PhysicsWeb. <http://physicsweb.org/articles/news/11/6/16/1>. Accessed 26 June 2007
Dissertation
Trent JW (1975) Experimental acute renal failure. Dissertation, University of California

Corrections and editing in response to comment 2.2: The suggested formatting has been applied consistently.

Corrections and editing in response to comment 2:

Comment 3: C. You MUST find a native English speaker to check your grammar and writing style in the revision. If you need our help, we can recommend an international writing company to support you with some fees.

Corrections and editing in response to comment 3: We have reviewed the entire text, reworked the abstract and conclusion, and made language and grammar corrections in the text. Final proof check is scheduled prior to print production. We plan to review the entire text with two native English speakers of academic grade – prior to print. Although we reviewed this version extensively, the final check would be to ensure that future editing and corrections are also reviewed and errors are corrected.
